# Hydrocele of the canal of Nuck: a case report with magnetic resonance hydrography findings

**DOI:** 10.1186/s40792-015-0086-5

**Published:** 2015-09-22

**Authors:** Rei Kono, Hiroshi Terasaki, Naotaka Murakami, Maki Tanaka, Jinryou Takeda, Toshi Abe

**Affiliations:** Department of Radiology, Japan Community Healthcare Organization (JCHO) Kurume General Hospital, 21 Kushiharamachi, Kurume, Fukuoka 830-0013 Japan; Department of Radiology, Kurume University School of Medicine, 67 Asahimachi, Kurume, Fukuoka 830-0011 Japan; Department of Surgery, Japan Community Healthcare Organization (JCHO) Kurume General Hospital, 21 Kushiharamachi, Kurume, Fukuoka 830-0013 Japan

**Keywords:** Hydrocele of the canal of Nuck, MRI, MR hydrography, Round ligament

## Abstract

Hydrocele of the canal of Nuck, also called the “female hydrocele,” is a rare developmental disorder in females. This entity is now believed to be more common now in comparison with previous reports; however, it is still an unfamiliar problem for physicians. The processus vaginalis accompanies the round ligament through the inguinal canal into the labium majus. This evagination of the parietal peritoneum forms the canal of Nuck in the female. The canal of Nuck normally loses its connection with the peritoneal cavity during the first year of life, but can result in a hernia or hydrocele when the connection of the canal of Nuck fails to close. Here, we present the case of a 43-year-old female who complained of swelling in the right inguinal region. Coronal and axial magnetic resonance imaging (MRI) revealed a cystic mass lesion with an irregular shape in the right inguinal region, and smaller cystic lesions extending alongside the right round ligament were also identified in the right side of the pelvic cavity. Magnetic resonance (MR) hydrography revealed the uninterrupted cystic lesion from the inguinal region to the pelvic cavity, with constrictions at the internal and external inguinal rings. These MR findings proved to be incredibly useful for surgical planning.

## Background

A hydrocele of the canal of Nuck is the female equivalent of a spermatic cord hydrocele in males; thus, this entity is called the “female hydrocele” [1, 2]. The processus vaginalis arises as an evagination of the parietal peritoneum. The seventeenth-century Dutch anatomist Anton Nuck described the processus vaginalis peritonei in the inguinal canal of a female and named it the canal of Nuck [1]. During embryogenesis, the processus vaginalis accompanies the round ligament or testis as it passes through the inguinal canal into the labium majus or scrotum, respectively [3]. The canal of Nuck normally undergoes complete obliteration during the first year of life, and its failure to do so may result in an inguinal hernia or a hydrocele. While preoperative diagnosis of this type of inguinal region hydrocele is important, the diagnosis of a hydrocele of the canal of Nuck is seldom made based on clinical findings alone [1]. In addition, this entity is unfamiliar to physicians, which is not mentioned enough in comprehensive surgical and gynecologic textbooks [2, 4, 5].

We present a case of a hydrocele of the canal of Nuck diagnosed with magnetic resonance (MR) hydrography, a useful technique that allowed us to evaluate the extension of the hydrocele from the inguinal region into the pelvic cavity.

## Case presentation

A 43-year-old female presented with swelling in the right inguinal region. The right inguinal bulge protruded with coughing and gradually became more conspicuous over the last 4 years. The painless bulge had persisted for 2 years prior to her presentation. On physical examination, a soft mass measuring approximately 4.0 cm in diameter was palpable in the right inguinal region. Patient’s vital signs and laboratory data were normal, with no history of local trauma noted.

**Fig. 1:**
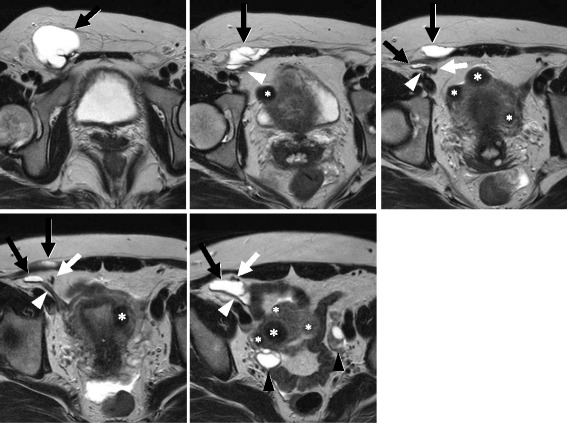
Axial T2-weighted MRI. Axial T2-weighted MRI shows a cystic mass (*black arrows*) measuring 48 × 37 mm in the right inguinal region. A smaller cystic lesion and fluid collection extend along the right round ligament in the pelvic cavity. Round ligament (*white arrowheads*), inferior epigastric artery and vein (*white arrows*), bilateral ovary (*black arrowheads*), and myomas (*asterisks*)

**Fig. 2:**
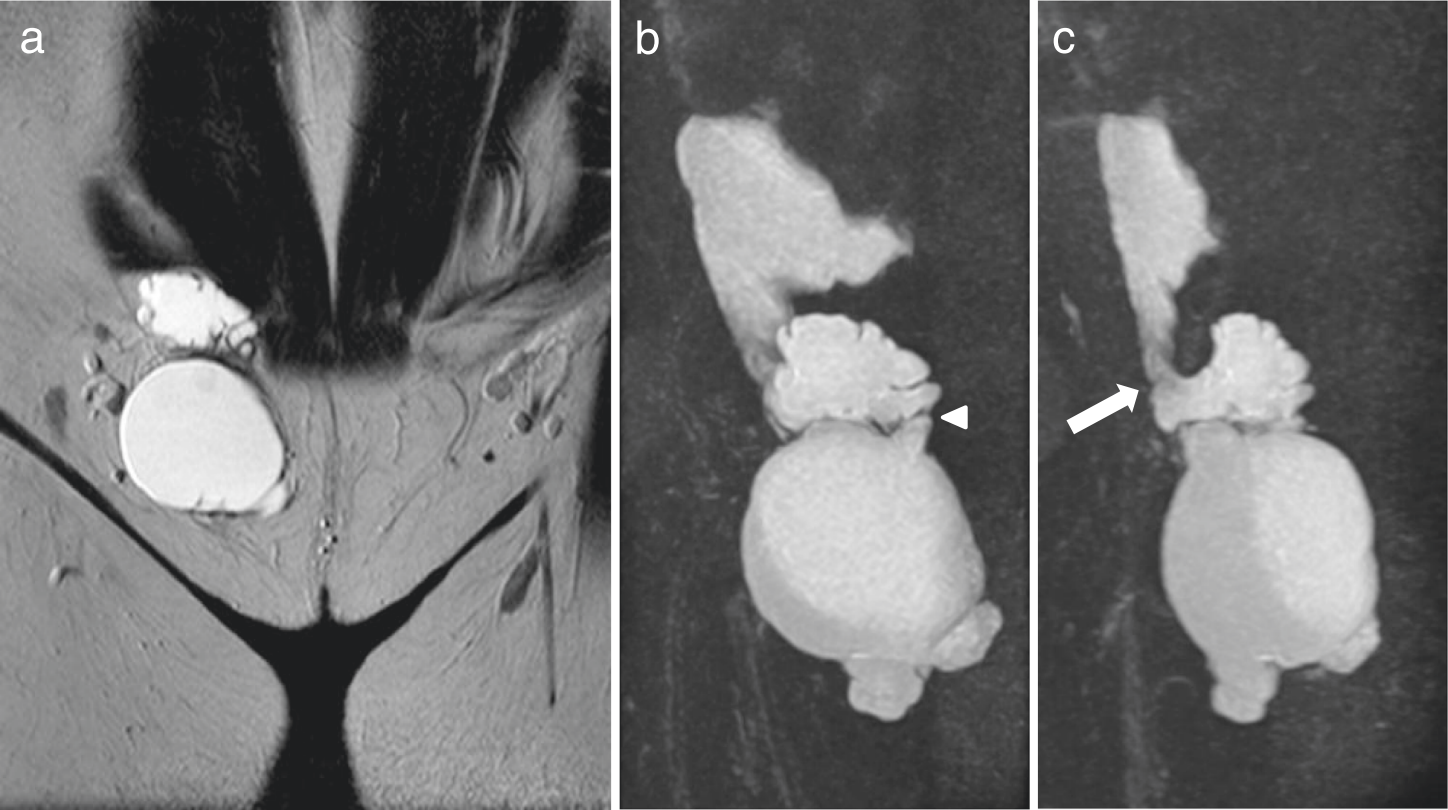
Coronal T2-weighted MRI and MR hydrography MIP images. Coronal T2-weighted MRI (**a**) and MIP images of MR hydrography [frontal view (**b**) and right anterior oblique view (**c**)] show an uninterrupted large cystic lesion extending from the pelvic cavity to the right inguinal region, measuring 13.5 cm in diameter with constrictions at the internal inguinal ring (*arrow*) and external inguinal ring (*arrowhead*)

MR imaging of the pelvis was performed with a 1.5-T magnetic resonance imaging (MRI) system (Gyroscan ACS NT; Philips Medical Systems, Germany). MR hydrography was performed using a SENSE body coil. A three-dimensional (3D) fast spin-echo imaging was applied as part of the comprehensive MR imaging examination was typically performed in patients. Parameters for 3D fast spin-echo imaging were as follows: repetition time/effective echo time, 3000 msec/721.7 msec; echo train length, 114; flip angle, 90°; section thickness, 2 mm; no gap between sections; field of view, 230 mm; matrix, 256 × 512; and acquisition time, 3 min 39 s. The MRI revealed an irregularly shaped cystic mass lesion measuring 48 × 37 mm in the right inguinal region, with a smaller cystic lesion and fluid collection evident in the right side of the pelvic cavity. These cystic lesions were isointense with muscles in the T1-weighted images and had marked high intensity on T2-weighted images. The T2-weighted images showed the cystic mass extending both within and outside the inguinal canal along the course of the right round ligament (Fig. 1). Neither intestinal nor omental components were identified in the cystic mass. Axial T2-weighted images showed multiple masses of low intensity in the uterus that were consistent with uterine myomas. Maximum intensity projection (MIP) imaging of MR hydrography showed that the right inguinal cystic mass lesion and the intrapelvic cystic lesion together formed an uninterrupted cystic lesion measuring 13.5 cm in diameter, with constrictions at the internal and external inguinal rings (Fig. 2). These findings seemed to indicate that the lesion was either a hydrocele of the canal of Nuck or endometriosis due to the minimal internal hemorrhagic component.

**Fig. 3:**
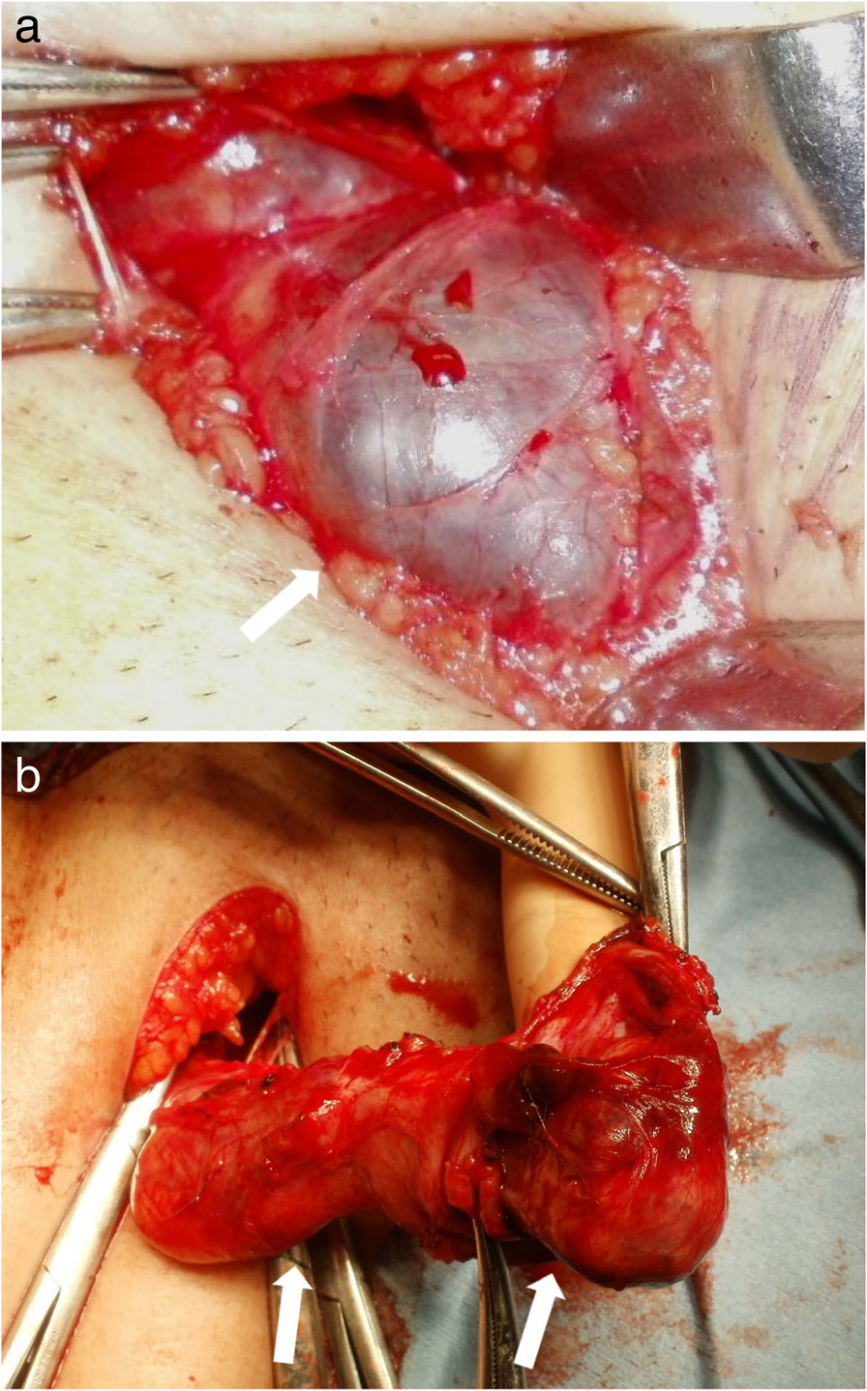
Intraoperative photograph. Surgery revealed a cystic lesion with a smooth (*arrow*), reddish surface, and minimal internal hemorrhage (**a**). High ligation at the internal inguinal ring and resection of the outside of the cystic lesion was performed. The lesion had adhered to the right round ligament of the uterus (**b**)

Surgery revealed that the cystic mass included a serous component extending from the right inguinal canal to the pubis adherent to the round ligament of the uterus (Fig. 3). High ligation at the internal inguinal ring and resection of the outside of the cystic lesion was performed; however, the adhered cystic lesion could not be dissected off from the round ligament, and a mesh plug repair was subsequently performed. The cystic lesion in the intrapelvic cavity remained after surgery. Pathological examination showed that the cystic lesion of irregular and fibrotic thick wall with a large number of small vessels had marked hemorrhages, histiocytic infiltration with phagocytosed hemosiderin, and inflammatory granulation tissue. Although a reddish mass lesion was found on the intracystic wall on gross pathologic examination, there was no evidence indicative of malignancy or endometriosis. These findings indicated that further surgery was not required for the remaining lesion, and a final diagnosis of hydrocele of the canal of Nuck was made. Postoperative recovery was uneventful, and the patient was eventually discharged. No recurrence of a mass in the inguinal region has occurred.

### Discussion

During embryonic development, the round ligament is derived from the gubernaculum, which is attached to the uterus near the origin of the fallopian tube. The cranial part of the gubernaculum becomes the ovarian ligament, whereas the caudal part forms the round ligament of the uterus. The round ligament runs through the internal ring, inguinal canal, and external ring, attaching terminally to the abdominal wall muscles [3]. The processus vaginalis accompanies the round ligament through the inguinal canal into the labium majus [6]. This evagination of the parietal peritoneum is the canal of Nuck in the female and corresponds to the processus vaginalis peritonei in the male. In males, this structure accompanies the spermatic cord into the inguinal canal before it reaches the scrotum. Normally, this peritoneal evagination undergoes complete obliteration during the first year of life [1]. When the processus vaginalis fails to close, the patient may develop a hernia or hydrocele. A hydrocele of the canal of Nuck, which is a collection of fluid within the processus vaginalis in females, is a rare entity, and it is analogous to a spermatic cord hydrocele [4–6].

Clinically, a hydrocele of the canal of Nuck can appear either as a painless or a moderately painful fluctuant inguinal mass, with no accompanying nausea or vomiting; therefore, it is not easy to diagnose this entity on clinical findings alone. These masses are not reducible and, if large enough, can be transilluminated. When the peritoneal evagination remains completely patent, it forms an avenue for an indirect inguinal hernia. Partial proximal obliteration, which leaves the distal portion of the processus vaginalis open, creates the anatomic prerequisite for a hydrocele of the canal of Nuck [4–6]. In adults, a hydrocele of the canal of Nuck should be first treated by surgical excision of the mass without puncturing it. Aspiration of a hydrocele of the canal of Nuck is inadequate and results in high recurrence rates. When the hydrocele is complicated by endometriosis, excision of both the mass and uterine round ligament is necessary [1, 4, 6].

The MRI findings for the canal of Nuck hydroceles have been reported for a few patients. In those reported by Park et al. [7] and Safak et al. [8], the hydrocele appeared as a thin-walled, tense cystic mass in the inguinal area. A hydrocele of the canal of Nuck has low intensity in T1-weighted images and high intensity in T2-weighted images. MRI reveals that these hydroceles extend both within and outside the inguinal canal and demonstrates the extension of the hydroceles very well, which allows for successful surgical excision. In the present case, the MRI revealed a cystic lesion, which extended along the round ligament from the pelvic cavity to the inguinal region. In addition, it enabled visualization of other internal structures which were neither omentum nor intestine in the cystic lesion; thus, this led us to a preoperative suspicion of a hydrocele of the canal of Nuck. Furthermore, the cystic lesion showed a slight increase in intensity compared with that of the muscles in the T1-weighted images. It is possible that this represented cystic lesions with minimal hemorrhagic changes. Although the cystic lesion was suspicious for a hydrocele of the canal of Nuck with endometriosis (so-called Nuck canal endometriosis), the histological examination revealed no evidence of endometriosis.

There are many causes of swelling in the female inguinal region, including inguinal hernia, tumors (lipoma, leiomyoma, and sarcoma), cysts, abscesses, and lymphadenopathy. When a hydrocele occurs in the vulva, the differential diagnosis should include a Bartholin gland cyst and a Gartner duct cyst. The hydrocele of the canal of Nuck is now believed to be more common than in previous reports; however, it is still an unfamiliar problem for physicians, and some cases are preoperatively misdiagnosed as inguinal hernias, Bartholin cysts, or Bartholin abscesses [2, 4, 5, 9]. It is important for clinicians, including surgeons and radiologists, to know and understand this entity accurately because many differential diagnoses are listed for inguinal masses in females. Ultrasonography is often used for the initial imaging of inguinal lesions. In previous reports, ultrasonographic findings of a hydrocele of the canal of Nuck have been described as a comma-shaped lesion with its tail directed toward the inguinal canal, a “cyst within a cyst” appearance, and a multicystic hydrocele. However, ultrasonography seems to be controlled by the capability of the specialist, and this examination lacks reproducibility. Moreover, there is a limited view and it is difficult to image the entirety of a large mass, as in this case, and to understand its relativity with surrounding tissues. Because MRI captures the same image, it is possible to share and understand the information. In addition, MR hydrography allows an easy grasp of the form of hydrocele from the liquid component, which is visualized as a high signal. Furthermore, it is necessary to understand the extension of the cystic lesion for proper surgical planning. MRI can be used effectively both as an imaging technique as well as a way to evaluate the extent of this condition. In particular, MR hydrography can identify the cystic mass form and can evaluate the excision range better and with more ease than other imaging modalities.

## Conclusions

We have presented a case of hydrocele of the canal of Nuck. MR hydrography revealed the uninterrupted cystic lesion extending from the inguinal region to the pelvic cavity, with constrictions at the internal and external inguinal rings. These MR findings proved to be incredibly useful for surgical planning.

## Consent

Written informed consent was obtained from the patient for publication of this case report and any accompanying images. A copy of the written consent is available for review by the Editor-in-Chief of this journal.

## Abbreviations

MR: magnetic resonance
MRI: magnetic resonance imaging
